# Clinical characteristics and risk factors of relative systemic hypertension and hypertension among sickle cell patients in Cameroon

**DOI:** 10.3389/fmed.2022.924722

**Published:** 2022-07-19

**Authors:** Arthemon Nguweneza, Valentina Josiane Ngo Bitoungui, Khuthala Mnika, Gaston Mazandu, Victoria Nembaware, Andre P. Kengne, Ambroise Wonkam

**Affiliations:** ^1^Division of Human Genetics, Department of Pathology, Faculty of Health Sciences, University of Cape Town, Cape Town, South Africa; ^2^Non-communicable Diseases Research Unit, South African Medical Research Council, Cape Town, South Africa; ^3^McKusick-Nathans Institute and Department of Genetic Medicine, Johns Hopkins University School of Medicine, Baltimore, MD, United States

**Keywords:** relative hypertension, hypertension, risk factors, sickle cell disease, Cameroon, Africa

## Abstract

Increased blood pressure (BP) has been associated with higher risk of stroke and mortality in Sickle Cell Disease (SCD). We investigated risk factors associated with Relative Systemic Hypertension (RSH) or systemic hypertension in SCD patients in Cameroon. Using R, Multivariate multinomial logistic regression modeling was used to examine the effects of the demographic, anthropometric, clinical, and laboratory factors to determine risk factors. A total of 815 individuals with SCD, including 380 (46.6%) males were analyzed. At baseline, the median age [interquartile range] was 18.0 [12.0–25.0] years, ranging from 3 to 66 years. Approximately three-quarters of the patients (*n* = 645; 79.1%) had normal BP, 151 (18.5%) had RSH and 19 (2.3%) had hypertension. Age (*P* < 0.001) and gender (*P* = 0.022) were significantly different across the BP categories. Weight (*P* < 0.001), height (*P* < 0.001), BMI (*P* < 0.001), pulse pressure (*P* = 0.020), history of stroke (*P* = 0.012), hemoglobin level (*P* = 0.002), red blood cell count (*P* = 0.031), creatinine (*P* < 0.001), and (estimated glomerular filtration rate) eGFR (*P* = 0.002) was also significantly different across the three BP categories. After adjustment, the significantly associated factors of RSH in the SCD patients were age [OR = 1.03, (95% CI = 1.01–1.06), *P* < 0.010], male gender [OR = 1.54, (95% CI = 1.04–2.27), *P* = 0.029], BMI [OR = 1.10, (95% CI = 1.04–1.17), *P* = 0.001]. After adjustment, the independent variables significantly associated factors of Hypertension in the SCD patients were age [OR = 1.05, (95% CI = 1.01–1.10), *P* = 0.034], male gender [OR = 3.31, (95% CI = 1.04–10.52), *P* = 0.042], BMI [OR = 1.14, (95% CI = 1.01–1.29), *P* = 0.027]. Creatinine was significantly associated with RSH [OR =1.31 (1.05–1.63), *P* = 0.016]. SCD patients with RSH or hypertension maybe at increased risk of renal dysfunction. We found relatively high prevalence of RSH and hypertension (20.8%) in SCD patients in Cameroon. Tailored Interventions that consider major risk factors (age, gender, and BMI) may lower BP pressure and prevent severe complications.

## Introduction

Sickle cell disease (SCD) patients, generally, have lower systolic, diastolic, and mean blood pressure compared to age and sex-matched controls (1, 2). There are no specific recommendations proposed regarding the defining criteria (and management) of hypertension in SCD patients. The lack of recommendations is a major concern since increased BP has been associated with higher risk of stroke and mortality in SCD patients, even in a range of systolic and diastolic BPs (SBP, DBP) that are considered relatively normal for the general population (i.e., lower than 140 mmHg) (2).

Blood pressure is a potential modulator of clinical severity in SCD patients, recent studies showed that relative systematic hypertension (RSH), defined as BP 120–139/70–89 mmHg, and Systemic Hypertension (BP >140/>90), considerably increased the risk of pulmonary hypertension and renal dysfunction (2). Previous studies have reported demographic, biological, anthropometric, and genetic factors to be associate with blood pressure in SCD patients (3–8). Blood pressure is a heritable trait with estimates of heritability indicating that 30–70% of the trait variance is attributable to genetic variation and a recurrent deleterious and loss of functions mutation with genes associated with lowering BP has been recently associate with long survival in SCD in Africa (9).

Identification of risk factors associated with BP variation in different populations is key to controlling BP, as well as preventing associated causes of mortality in SCD patients. We investigated risk factors associated with RSH or systemic hypertension in SCD patients in Cameroon to gain insight into the pathophysiology of BP variation in this disease in an African setting.

## Patients and methods

### Ethical approval

A proposal was submitted to the University of Cape Town, Faculty of Health Sciences Human Research Ethics Committee, Cape Town, South Africa (HREC/REF: R015/2018). All patients older than 18 years signed consent forms, while informed consent was given by the parents or guardians for participants younger than 18 years old, in accordance with the Declaration of Helsinki. This study was approved by the National Ethical Committee of the Ministry of Public Health of Cameroon (No 193/CNE/SE/15).

Written and signed informed consent forms were obtained from adult participants and parents/guardians of minor patients. An assent was also obtained from the participants of more than 7 years old.

### Participants' recruitment

All SCD patients with complete socio-demographic, clinical, laboratory variables, and complete systolic and diastolic blood pressure measurements were included in the study. The data were obtained from a cross-sectional study conducted in Cameroon from May 2016 to July 2018. The data were collected from nine hospitals from five cities in Cameroon, including Yaoundé, Douala, Bafoussam, Bertoua, and Maroua. Patients who have not experienced a painful crisis a month before, and who had not received a blood transfusion in the past 6 months, were recruited irrespective of age and gender.

### Use of variables

#### Dependent variables

Sickle cell disease patients with a SBP within the range of 120–139 mmHg and/or DBP within the range of 80–89 mmHg is defined as having RSH. Systemic hypertension is further defined as SBPs greater than 140 mmHg or DBPs greater than 90 mmHg. Participants who had incomplete/out of range blood pressure readings were excluded from the analysis.

#### Independent variables

Information on demographics, including age, residential location, sex, ethnicity, educational level, marital status, and household income status, was collected using a standard questionnaire involving the household and individual levels. Clinical information and laboratory information were also collected. Those who had incomplete/out of range relevant information such as age, gender, BMI, demographic, clinical, laboratory information were also excluded from the dataset.

### Statistical analysis

All our analysis was analyzed using R (version 4.0.2). Continuous variables were presented as median and interquartile range (IQR) and categorical variables as percentages (%).

Categorical variables were compared using *X*^2^-test or Fisher exact test if the expected count in a cell was less than five while continuous variables were compared according to BP category with the Kruskal–Wallis test.

Multivariate multinomial logistic regression modeling was used to examine the effects of the demographic, anthropometric, clinical, and laboratory factors to determine the potential independent risk factors for RSH and Systemic hypertension.

A final model was created that included all the predictors and interactions that were significantly associated at the level of *P* < 0.05. The findings presented as crude and adjusted odds ratios with their 95% confidence intervals (CI).

## Results

### Baseline characteristics

Table 1 Shows the demographic, anthropometric, clinical and laboratory characteristics of the BP categories. Our analysis included 815 individuals with SCD, of whom 645 (79%) had normal BP, 151 (19%) had RSH, 19 (2%) had systemic hypertension. 380 (46.6%) were males. At baseline, the median age [interquartile range] was 18.0 [12.0–25.0] years, ranging from 3 to 66 years. Approximately three-quarters of the patients (645 or 79.1%) were normal BP, 151 (18.5%) had relative hypertension and 19 (2.3%) had hypertension. Age (*P* < 0.001) and gender (*P* = 0.022) were significantly different across the BP categories, with age increasing with BP. Weight (*P* < 0.001), height (*P* < 0.001), BMI (*P* < 0.001), pulse pressure (*P* = 0.020), history of stroke (*P* = 0.012), hemoglobin (*P* = 0.002), red blood cell count (*P* = 0.031), creatinine (*P* < 0.001), and eGFR (*P* = 0.002) were also significantly different across the three BP categories.

**Table 1:** Baseline demographic, anthropometric, clinical and laboratory characteristics of Cameroonian SCD patients by BP levels.

**Characteristics**	**All (*n*/*N*, %)**	**Normal (*n* =, %)**	**RSH (*n* = , %)**	**Hypertension (*n* = , %)**	***P*-value**
		**or median (IQR)**	**or median (IQR)**	**or median (IQR)**	
**Demographics**
Age, years	815/815 (100.0)	17.0 (11.0–24.0)	22.0 (18.0–28.0)	24.0 (18.0–40.5)	<0.001
Aged less than 18	373/815 (45.8)	336/645 (52.0)	33/151 (22.0)	4/19 (21.1)	<0.001
Aged older or equal 18	442/815 (54.2)	309/645 (48.0)	118/151 (78.0)	15/19 (78.9)	
Gender, male	380/815 (46.6)	289/645 (44.7)	77/151 (51.3)	14/19 (73.7)	0.022
**Anthropometric and clinical**
Weight	815	46.0 (30.0–55.0)	56.0 (50.0–62.0)	60.0 (50.0–68.0)	<0.001
Height	815	1.58 (1.37–1.67)	1.67 (1.60–1.73)	1.64 (1.58–1.77)	<0.001
Body mass index (BMI)	815	18.0 (16.0–20.0)	20.0 (18.0–22.0)	21.0 (19.5–23.0)	<0.001
Pulse pressure^a^	789	91.0 (81.3–101)	88.0 (80.3–96.8)	84.0 (76.0–92.5)	0.020
History of stroke^a^	28/803 (3.5)	20/637 (3.1)	5/147 (3.4)	3/19 (15.8)	0.012
History of kidney disease^a^	82/807 (10.2)	69/640 (10.8)	10/148 (6.8)	3/19 (15.8)	0.246
History of Pulmonary hypertension^a^	68/807 (8.4)	52/640 (8.1)	13/148 (8.8)	3/19 (15.8)	0.4888
History of transfusion^a^	634/813 (78.0)	504/644 (78.3)	118/150 (78.7)	12/19 (63.2)	0.286
Hydroxuria^a^	72 /807 (8.9)	58/640 (9.1)	14/148 (9.5)	0/19 (0.0)	0.381
**Biological data**
Hemoglobin (g/dl)	797/815	7.60 (6.80–8.50)	8.00 (7.10–8.90)	8.10 (7.80–10.8)	0.0020
Hemoglobin F (%)	794/815	6.40 (3.80–11.6)	6.30 (3.40–11.5)	8.35 (4.73–12.3)	0.459
White blood cell count (10^9^/L)	797/815	10.3 (7.80–13.0)	9.80 (7.77–12.6)	9.45 (8.30–11.3)	0.637
Mean corpuscular volume (fl)	798/815	88.0 (82.0–95.0)	89.0 (83.0–95.0)	85.0 (77.5–92.0)	0.360
Red blood cell count	794/815	2.70 (2.30–3.13)	2.83 (2.40–3.19)	2.87 (2.63–3.38)	0.031
Creatinine (mg/dl)	770/815	0.45 (0.37–0.60)	0.60 (0.40–0.78)	0.65 (0.50–0.87)	<0.001
(Estimated glomerular filtration rate) eGFR	760/815	175 (151–204)	158 (136–178)	150 (119–182)	0.002

a *Total number of children may differ because of missing data; IQR, interquartile range*.

### Univariate and multivariate analysis

#### The normal BP group vs. RSH group

Among SCD patients, univariate analyses indicated that these variables were significantly more common risk factors for higher BP values among patients with RSH than those with normal BP: Age (*P* < 0.001), patients >18 years (*P* < 0.001), weight (*P* < 0.001), height (*P* < 0.001), BMI (*P* < 0.001), pulse pressure (*P* = 0.046), creatinine (*P* < 0.001), eGFR (*P* < 0.001) and hemoglobin (*P* = 0.020) (Table 2). Multivariate analyses found that age [OR = 1.02, (95% CI = 1.01–1.05), *P* = 0.021], creatinine [OR = 1.310, 95% CI = 1.05–1.63, *P* = 0.016], BMI [OR = 1.09, (95% CI = 1.03–1.16), *P* = 0.002] were independent risk factors for high BP values in SCD patients with RSH compared with SCD patients with normal BP values (Table 3).

**Table 2:** Univariate multinomial logistic regression analyses of factors associated with RSV and hypertension among SCD patients in Cameroon (reference: Normal BP).

**Factors**	**RSH vs. normal**		**Hypertension vs. normal BP**	
	**cOR (95% CI)**	***P*-value**	**cOR (95% CI)**	***P*-value**
**Demographics**
Age^*^	1.05 (1.03–1.06)	<0.001	1.08 (1.04–1.12)	<0.001
Aged less than 18 (ref.)				
Aged older or equal 18^*^	3.89 (2.56–5.89)	<0.001	4.07 (1.34–12.41)	0.013
Gender, female (ref.)				
Gender, male^*^	1.28 (0.89–1.83)	0.170	3.45 (1.22–9.69)	0.019
**Anthropometric and clinical**
Weight^*^ (kg)	1.05 (1.04–1.07)	<0.001	1.07 (1.03–1.11)	<0.001
Height (m)^*^	76.1 (21.77–265.89)	<0.001	15.9 (0.96–264.38)	0.054
Body mass index^*^	1.16 (1.10–1.23)	<0.001	1.22 (1.11–1.35)	<0.001
Pulse pressure^*^	0.98 (0.97–1.00)	0.046	0.95 (0.92–1.00)	0.019
History of stroke^*^ vs. No (ref)	1.03 (0.38–2.77)	0.957	5.59 (1.51–20.66)	0.010
History of kidney disease vs. No (ref)	0.60 (030–1.19)	0.145	1.57 (0.44–5.54)	0.480
History of pulmonary hypertension vs. No (ref)	1.11 (058–2.10)	0.749	2.20 (0.26–7.78)	0.223
History of transfusion vs. No (ref)	1.00 (0.65–1.54)	0.985	0.47 (0.18–1.23)	0.128
Hydroxuria	1.00 (0.53–1.89)	0.992	0.00 (−7.07–1.7)	0.762
**Biological data**
Hemoglobin (g/dl)^*^	1.12 (1.01–1.23)	0.020	1.36 (1.14–1.61)	<0.001
Hemoglobin F (%)	0.99 (0.97–1.03)	0.746	1.04 (0.97–1.11)	0.226
White blood cell count (10^9^/L)	0.99 (0.95–1.09)	0.782	0.95 (0.85.1.08)	0.485
Mean corpuscular volume (fl)	1.00 (1.00–1.02)	0.528	1.00 (0.94–1.01)	0.062
Red blood cell count^*^	1.23 (1.00–1.53)	0.061	1.9 (1.24–2.94)	0.03
Creatinine (mg/dl)	1.24 (1.15–1.34)	<0.001	1.38 (1.17–1.62)	<0.001
eGFR	1.00 (0.98–1.00)	<0.001	0.99 (0.98–1.00)	0.007

*cOR, Crude odds ratio; CI, Confidence interval*;

* *denotes significant at the 5% level*.

**Table 3:** Multivariate multinomial logistic regression analyses of factors associated with RSV and hypertension among SCD patients in Cameroon (reference: Normal BP).

**Factors**	**RSH vs. normal BP**		**Hypertension vs. normal BP**	
	**aOR (95% CI)**	***P*-value**	**aOR (95% CI)**	***P*-value**
Age, years^*^	1.02 (1.01–1.05)	0.021	1.05 (1.01–1.10)	0.034
Gender, male vs. female (ref.)^*^	1.20 (1.10–1.80)	0.372	3.31 (1.04–10.52)	0.042
Body mass index (BMI)^*^	1.09 (1.03–1.10)	0.002	1.14 (1.01–1.29)	0.027
Pulse pressure	1.00 (0.98–1.01)	0.679	0.98 (0.93–1.01)	0.289
History of stroke vs. No (ref)	0.89 (0.30–2.56	0.830	2.24 (0.42–11.79)	0.339
Creatinine (mg/dl)	1.31 (1.05–1.63)	0.016	1.26 (0.75–2.09)	0.373
Hemoglobin (g/dl)	1.00 (0.70–1.40)	0.234	0.88 (0.35–2.47)	0.572
Red blood cell count	1.02 (0.87–1.13)	0.684	1.15 (0.81–2.13)	0.145

*aOR, Adjusted odds ratio; CI, Confidence interval*;

* *denotes significant at the 5% level*.

#### The normal BP group vs. systemic hypertension group

Among SCD patients, univariate analyses indicated that these variables were significantly more common risk factors for higher BP values among SCD patients with hypertension than those SCD patients with normal BP: Age (*P* < 0.001), patients >18 years (*P* = 0.013), male gender (*P* = 0.019), weight (*P* < 0.001), BMI (*P* < 0.001), pulse pressure (*P* = 0.019), hemoglobin (*P* < 0.001), creatinine (*P* < 0.001) and Red blood cell count (*P* = 0.03) (Table 2). Multivariate analyses found that age [OR = 1.05, (95% CI = 1.01–1.10), *P* = 0.034], male gender [OR = 3.31, (95% CI = 1.04–10.52), *P* = 0.042], BMI [OR = 1.14, (95% CI = 1.01–1.29), *P* = 0.027] were independent risk factors for higher BP values in SCD patients with hypertension compared with SCD patients with normal BP values (Table 3).

**Figure 1:**
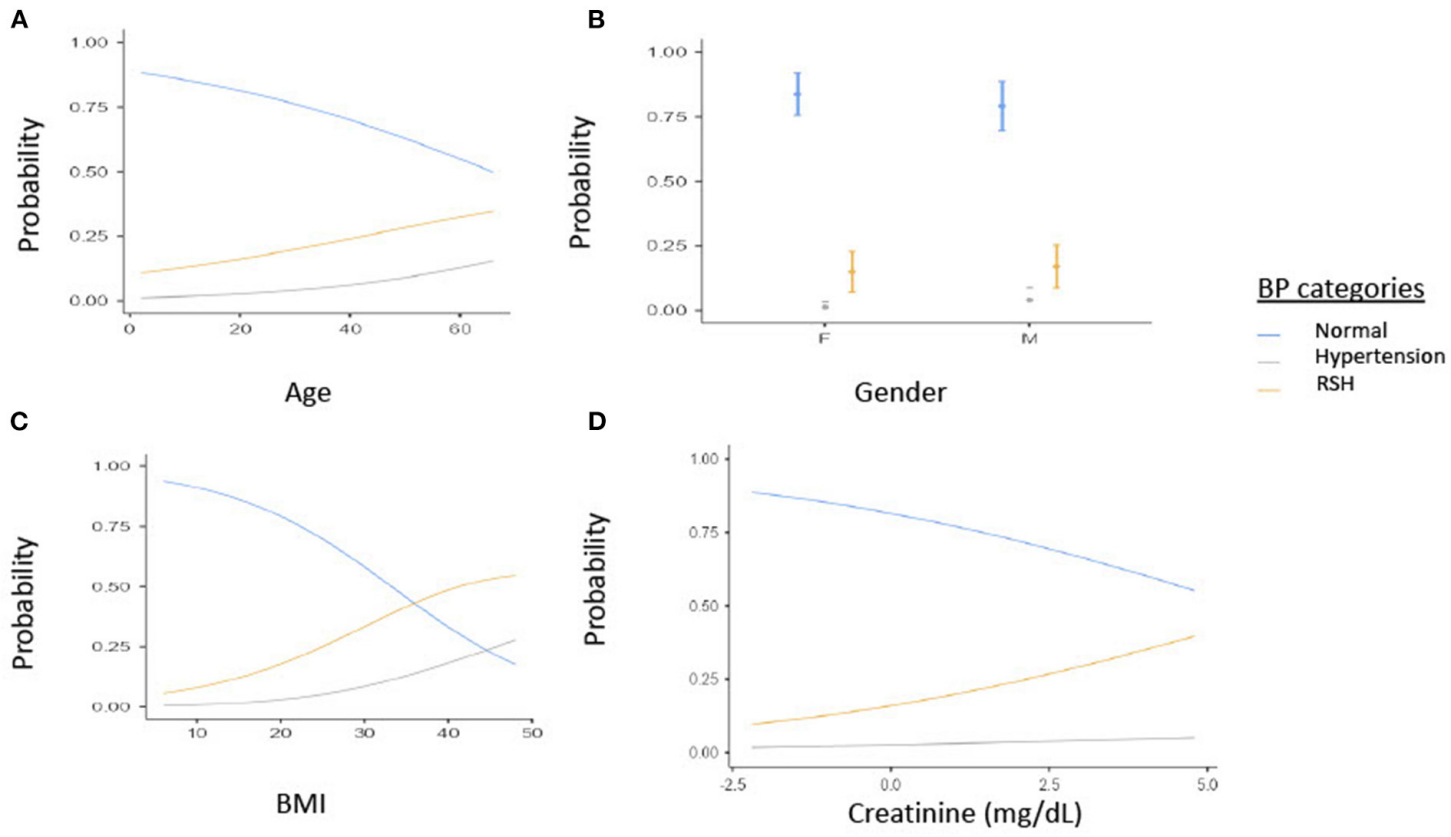
Estimated marginal means of **(A)** Age, **(B)** Gender, **(C)** BMI by BP categories, **(D)** creatine (mg/dl). BP, Blood pressure; BMI, Body mass index; RSH, Relative systemic hypertension; F, Females; M, Males.

Additionally, Figure 1 Illustrates the relationship between BP and age, gender, BMI. As age increases the probability of SCD patients having RSH or hypertension increases (Figure 1A). Secondly, Males have a higher probability of having RSH or hypertension than females among SCD patients (Figure 1B). For every increase in BMI units, the probability of having RSH or Hypertension increases among SCD patients (Figure 1C). Lastly, for every increase in creatine units, the probability of having RSH or Hypertension increases among SCD patients (Figure 1D).

## Discussion

This study determined the role of demographic, anthropometric, clinical and laboratory factors associated with RSH and hypertension among SCD patients in Cameroon, one of the rare attempts from Africa. The main findings, from this relatively large dataset are as follows. Approximately one quarter of our 815 SCD patients were classified in either RSH or systemic hypertension group. At baseline, we observed statistically significant differences in age, gender, weight, height, BMI, pulse pressure, a history of stroke, hemoglobin, and red blood cell count across our three BP groups (normal BP, RSH, hypertension). We found that age, BMI, creatinine, and male gender were independently associated with an increased risk of RSH and systemic hypertension after adjusting for other variables.

The nearly 19% prevalence of RSH reported in this study was similar to that of 17% reported in studies from North America by Becker et al. (10) and Bodas et al. (11). In similar setting as our study, RSH was lower to that of 45% reported by Benneh Akwasi Kuma et al. (12) and 44% found by Makubi et al. (13), the participants in these studies were adult patients, whereas our study included both pediatric and adult patients. The 2% prevalence of systemic hypertension reported in this study from Cameroon also agrees with previous reports from both high and low incomes settings, which have reported the prevalence of systemic hypertension in SCD patients to be lower than that of the general population (2–8% vs. 28%, respectively) (13–15). Potential explanations of low prevalence of RSH and systemic hypertension in SCD patients include Sodium and water wasting due to the medullary defect, (16) systemic vasodilatation compensating for microcirculatory flow disturbances, (14) increased production of prostaglandins and nitric oxide, (17) reduced vascular reactivity, (16) and pre-mature deaths that remove those individuals whose BP might reach hypertensive levels in middle adulthood (13).

Unsurprisingly, this study also found that age was significantly associated with BP in SCD patients, SCD patients with RSH and systemic hypertension were older than SCD patients with normal BP values suggesting that advancing age contributed to their higher BP values. This finding corroborates with previous reports in developed countries; (1, 8) and in Africa (18), that reported that BP rapidly increases with advancing age in SCD patients starting in the early twenties. With the improved survival of patients with SCD patients, the incidence of RSH or systemic hypertension is expected to rise, thus screening and awareness are necessary to prevent the expected complications, in all part of the world. Indeed, mortality in adult with SCD in the USA and other high-income countries have not changed over the past four decades, mostly dues to debilitating and severe cardiovascular complications (19). Most of the previous data is from 18-year olds. However, younger patients may already show elevated BP and risk for complications.

Pegelow et al. (1) demonstrated that BP values were higher in males than in females, which is consistent with our results showing that male gender is independently associated with RSH and systemic hypertension in SCD patients. This gender disparity in BP is likely due to gender-related differences in SCD biology or health-seeking behavior between genders (20, 21). For instance, older males with elevated BP relative to the SCD population are at increased risk of stroke than age-matched females (12). SCD males have higher pulse pressure, a predictor of all-cause mortality, than age-matched SCD female patients (12, 22) which further highlights the risk of adverse outcomes associated with RSH and systemic hypertension in males. Another study suggests that regular medical visits are critical for improving hypertension awareness among young adults and reducing gender disparities in cardiovascular health (21).

Consistent with previous studies, Oguanobi et al. (23) in Nigeria, and Pegelow et al. (1) who reported that BMI correlates positively with SBP and DBP and Homi et al. who reported that low weight is a risk factor for low BP. In this study, we found that BMI correlates positively with BP, and BMI was independently associated with RSH and systemic hypertension among SCD patients. Suggesting that a higher BMI in SCD patients with RSH or systemic hypertension may contribute to their higher BP values compared to the SCD patients with normal BP. SCD patients have lower BMI compare to general population but increased BMI in SCD patients has potential to modulate BP (8). In addition, the prevalence of obesity in patients with SCD seems to be on the increase. Obesity is a risk factor for other diseases, including, but not limited to, type 2 diabetes, hypertension, sleep apnea, cardiovascular disease (24). These diseases, in turn, worsen the clinical picture of SCD and increase the frequency of vaso-occlusive crises (VOCs) (24). Because of the clinical importance as well as public health importance of RSH or systemic hypertension, the ability to identify otherwise normal BMI is of paramount importance, particularly in SCD patients.

Furthermore, measuring BMI alone, in SCD, is sufficient to screen for adiposity and obesity. Previous reports show the body composition of SCD patients with normal mean BMI (22.6 kg/m^2^), showed a 32.6% fat composition, indicating high levels of adiposity. Since fat accumulation and adipocyte secretion are responsible for many hormonal changes playing a role in the development of vascular dysfunction and hypertension in the general population, this could be the case in SCD patients too, even if BMI values are normal. Therefore, further studies are needed to better understand the relationship between BMI; hormonal status and BP in SCD.

Previous studies reported that SCD patients with SBP 120–139 mm Hg or DBP 70–89 mm Hg had elevated levels of creatinine compared to SCD patients with SBP <120 mm Hg and DBP <70 mm Hg (2). In this study we found that creatinine was independently associated with RSH. Additionally, SCD patients in RSH and systemic hypertension group had a higher creatinine compared to SCD patients in the normal group. Suggesting that SCD patients with RSH or hypertension are at increased risk of renal dysfunction. Longitudinal studies are needed to better understand temporal relationship between renal dysfunction and RSH.

Previous studies found Increasing hemoglobin, blood viscosity and blood transfusion to be independent risk factors for RSH or hypertension in SCD patients. However, in this study we did not find these factors to be significantly associated with BP among SCD patients (25). These observed differences may be explained by differences in study design, patient's clinical characteristics and thresholds used to define RSH or systemic hypertension.

- Our participants were recruited form referral hospitals. Thus, the findings may not be representative of RSH or systemic hypertension seen in a community. Nevertheless, our analysis is based on the large and well-characterized homozygous study population in a resource-limited country. Therefore, these findings expand the understanding of risk factors for RSH and systemic hypertension in SCD beyond what has been reported from resource-limited settings.
- The exclusion of incomplete records with missing BP might have introduced some bias. Additionally, BP was measured at single time point which might have increased some patients' likelihood of developing white coat hypertension. Previous studies have highlighted the importance of 24-h ambulatory blood pressure monitoring in diagnosing masked hypertension (26).
- The inability to follow up the cohort as a longitudinal study is a limitation.

In conclusion, this study found evidence of the prevalence of RSH and hypertension in the SCD patients in Cameroon. Age, male gender, BMI was found to be independently associated factors of RSH and hypertension in the SCD patients in Cameroon. Tailored Interventions that consider these risk factors have potential to lower BP pressure in SCD patients and prevent developing severe complications.

## Data availability statement

The raw data supporting the conclusions of this article will be made available by the authors, without undue reservation.

## Ethics statement

A proposal was submitted to the University of Cape Town, Faculty of Health Sciences Human Research Ethics Committee, Cape Town, South Africa (HREC/REF: R015/2018). All patients older than 18 years signed consent forms, while informed consent was given by the parents or guardians for participants younger than 18 years old, in accordance with the Declaration of Helsinki. This study was approved by the National Ethical Committee of the Ministry of Public Health of Cameroon (No 193/CNE/SE/15). Written and signed informed consent forms were obtained from adult participants and parents/guardians of minor patients. An assent was also obtained from the participants of more than 7 years old. Written informed consent to participate in this study was provided by the participants' legal guardian/next of kin.

## Author contributions

AW conceived the study. AN, VN, GM, and AW made substantial contributions to the conception, design of the work, methodology, analysis, data interpretation, and wrote the final manuscript. AN and GM analyzed and interpreted the data. AN issued the first draft of the paper. AN, VN, KM, GM, VN, AK, and AW critically revised successive drafts of the manuscript. VN, GM, AK, and AW supervised the project and compiled the revisions. All authors have read and agreed to the published version of the manuscript.

## Funding

This work was supported by the National Heart, Lung, and Blood Institute of the National Institutes of Health (Award Number U24HL135600) and (Award Number 1U24HL135881).

## Conflict of interest

The authors declare that the research was conducted in the absence of any commercial or financial relationships that could be construed as a potential conflict of interest.

## Publisher's note

All claims expressed in this article are solely those of the authors and do not necessarily represent those of their affiliated organizations, or those of the publisher, the editors and the reviewers. Any product that may be evaluated in this article, or claim that may be made by its manufacturer, is not guaranteed or endorsed by the publisher.

## References

[1] Pegelow CH Colangelo L Steinberg M Wright EC Smith J Phillips G. Natural history of blood pressure in sickle cell disease: risks for stroke and death associated with relative hypertension in sickle cell anemia. Am J Med. (1997) 102:171–7. 10.1016/S0002-9343(96)00407-X 9217567

[2] Gordeuk VR Sachdev V Taylor J Gladwin MT Kato G Castro OL. Relative systemic hypertension in patients with sickle cell disease is associated with risk of pulmonary hypertension and renal insufficiency. Am J Hematol. (2008) 83:15–18. 10.1002/ajh.21016 17696198 PMC3398810

[3] Lamarre Y Lalanne-Mistrih M Romana M Lemonne N Mougenel D Waltz X. Male gender, increased blood viscosity, body mass index and triglyceride levels are independently associated with systemic relative hypertension in sickle cell anemia. PLoS ONE. (2013) 8:e66004. 10.1371/journal.pone.0066004 23785465 PMC3681937

[4] Bhatnagar P Barron-Casella EJ Bean C Milton JN Baldwin CT Steinberg MH. Genome-wide meta-analysis of systolic blood pressure in children with sickle cell disease. PLoS ONE. (2013) 8:e74193. 10.1371/journal.pone.0074193 24058526 PMC3772989

[5] Campbell K Asnani M Cuningham-Myrie C Cumming V Barton EN Reid M. Determinants of blood pressure in adults with sickle cell disease. West Indian Med J. (2007) 56:95. 10.1590/S0043-31442007000300029

[6] Novelli EM Hildesheim M Rosano C Vanderpool R Simon M Kato GJ. Elevated pulse pressure is associated with hemolysis, proteinuria and chronic kidney disease in sickle cell disease. PLoS ONE. (2014) 9:e114309. 10.1371/journal.pone.0114309 25478953 PMC4257593

[7] Lemonne N Romana M Lamarre Y Hardy-Dessources MD Lionnet F Waltz X. Association between relative systemic hypertension and otologic disorders in patients with sickle cell-hemoglobin C disorder. Am J Hematol. (2014) 89:667–667. 10.1002/ajh.23717 24668790

[8] Desai PC Deal AM Brittain JE Jones S Hinderliter A Ataga KI. Decades after the cooperative study: a re-examination of systemic blood pressure in sickle cell disease. Am J Hematol. (2012) 87:65–8. 10.1002/ajh.23278 22718523 PMC3456969

[9] Wonkam A Chimusa ER Mnika K Pule GD Ngo Bitoungui VJ Mulder N. Genetic modifiers of long-term survival in sickle cell anemia. Clin Transl Med. (2020) 10:e152. 10.1002/ctm2.152 32898326 PMC7423184

[10] Becker AM Goldberg JH Henson M Ahn C Tong L Baum M. Blood pressure abnormalities in children with sickle cell anemia. Pediatr Blood Cancer. (2014) 61:518–22. 10.1002/pbc.24843 24424792

[11] Bodas P Huang A O'Riordan MA Sedor JR Dell KM. The prevalence of hypertension and abnormal kidney function in children with sickle cell disease: a cross-sectional review. BMC Nephrol. (2013) 14:237. 10.1186/1471-2369-14-237 24168027 PMC4231610

[12] Benneh-Akwasi Kuma A Owusu-Ansah AT Ampomah MA Sey F Olayemi E Nouraie M. Prevalence of relative systemic hypertension in adults with sickle cell disease in Ghana. PLoS ONE. (2018) 13:e0190347. 10.1371/journal.pone.0190347 29300776 PMC5754083

[13] Makubi A Mmbando BP Novelli EM Lwakatare J Soka D Marik H. Rates and risk factors of hypertension in adolescents and adults with sickle cell anemia in Tanzania: 10 years' experience. Br J Haematol. (2017) 177:930–7. 10.1111/bjh.14330 27650269 PMC5612392

[14] Maaten JC Serné EH Bakker SJ van Eps WS Donker AJ Gans RO. Effects of insulin on glucose uptake and leg blood flow in patients with sickle cell disease and normal subjects. Metabolism. (2001) 50:387–92. 10.1053/meta.2001.21681 11288031

[15] Bruno D Wigfall DR Zimmerman SA Rosoff PM Wiener JS. Genitourinary complications of sickle cell disease. J Urol. (2001) 166:803–11. 10.1016/S0022-5347(05)65841-7 11490223

[16] Hatch FE Crowe LR Miles DE Young JP Portner ME. Altered vascular reactivity in sickle hemoglobinopathy. A possible protective factor from hypertension. Am J Hypertens. (1989) 2:2–8. 10.1093/ajh/2.1.2 2643968

[17] Allon M Lawson L Eckman JR Delaney V Bourke E. Effects of non-steroidal anti inflammatory drugs on renal function in sickle cell anemia. Kidney Int. (1988) 34:500–6. 10.1038/ki.1988.209 3199668

[18] Akingbola TS Tayo BO Salako B Layden JE Hsu LL Cooper RS. Comparison of patients from Nigeria and the USA highlights modifiable risk factors for sickle cell anemia complications. Hemoglobin. (2014) 38:236–43. 10.3109/03630269.2014.927363 24941131 PMC4161131

[19] Chaturvedi S DeBaun MR. Evolution of sickle cell disease from a life-threatening disease of children to a chronic disease of adults: the last 40 years. Am J Hematol. (2016) 91:5–14. 10.1002/ajh.24235 26547630

[20] Ceglie G Di Mauro M Tarissi De Jacobis I de Gennaro F Quaranta M Baronci C. Gender-related differences in sickle cell disease in a pediatric cohort: a single-center retrospective study. Front Mol Biosci. (2019) 6:1–5. 10.3389/fmolb.2019.00140 31867340 PMC6906547

[21] Everett B Zajacova A. HHS public access. Front Mol Biosci. (2016) 61:1–17. 10.1080/19485565.2014.929488 25879259 PMC4896734

[22] Pikilidou M Yavropoulou M Antoniou M Papakonstantinou E Pantelidou D Chalkia P. Arterial stiffness and peripheral and central blood pressure in patients with sickle cell disease. J Clin Hypertens. (2015) 17:726–31. 10.1111/jch.12572 25991400 PMC8031914

[23] Oguanobi NI Onwubere BJC Ibegbulam OG Ike SO Anisiuba BC Ejim EC. Arterial blood pressure in adult Nigerians with sickle cell anemia. J Cardiol. (2010) 56:326–31. 10.1016/j.jjcc.2010.07.001 20727714

[24] Woods KF Ramsey LT Callahan LA Mensah GA Litaker MS Kutlar A. Body composition in women with sickle cell disease. Ethn Dis. (2001) 11:30–5. https://pubmed.ncbi.nlm.nih.gov/11289248/11289248

[25] Nguweneza A Oosterwyk C Banda K Nembaware V Mazandu G Kengne AP. Factors associated with blood pressure variation in sickle cell disease patients: a systematic review and meta-analyses. Expert Rev Hematol. (2022) 15:359–68. 10.1080/17474086.2022.2043743 35209795 PMC12086708

[26] Shatat IF Jakson SM Blue AE Johnson MA Orak JK Kalpatthi R. Masked hypertension is prevalent in children with sickle cell disease: a Midwest pediatric nephrology consortium study. Pediatr Nephrol. (2013) 28:15–120. 10.1007/s00467-012-2275-9 22886281

